# Analysis of m6A RNA methylation in *Caenorhabditis elegans*

**DOI:** 10.1038/s41421-020-00186-6

**Published:** 2020-07-14

**Authors:** Erdem Sendinc, David Valle-Garcia, Alan Jiao, Yang Shi

**Affiliations:** 1 Division of Newborn Medicine and Epigenetics Program, Department of Medicine, Boston Children’s Hospital, Boston, MA 02115 USA; 2 Department of Cell Biology, Harvard Medical School, Boston, MA 02115 USA; 3 Present Address: Departamento de Medicina Molecular y Bioprocesos, Instituto de Biotecnologia, Universidad Nacional Autonoma de Mexico, Cuernavaca, Morelos 62210 Mexico

**Keywords:** RNA modification, Ribosome

Dear Editor,

One of the most abundant RNA modifications is N^6^-methyladenosine (m6A), which is present in RNA from all forms of life, including viruses. This modification has been detected in many types of RNAs, such as mRNA, ribosomal RNA, long non-coding RNAs, small nuclear RNAs, and microRNAs. Diverse sets of proteins have been characterized that methylate, demethylate, and specifically bind to this modification in different organisms.

*Caenorhabditis elegans* is a unique model organism with abundant m6A modification, although its genome does not code for orthologs of the well-characterized m6A methyltransferase METTL3/METTL14 complex or the demethylases FTO and ALKBH5. Furthermore, orthologs of the YTH family of m6A reader proteins seem to be absent from the worm genome as well. To gain insights into how this modification is installed in this organism, we set out to identify enzymes that contribute to the abundant level of m6A in *C. elegans*. We designed a targeted RNAi screen by which the expression of 22 candidate RNA methyltransferase genes were knocked down. We measured global RNA methylation level by HPLC–MS/MS analysis after two generations of RNAi-mediated knock down. The knock down of two candidate methyltransferases resulted in a decrease in global m6A level in total RNA (Supplementary Fig. S1). The first methyltransferase, F33A8.4, is an ortholog of the human ZCCHC4 gene (Supplementary Fig. S2). This conserved methyltransferase has recently been shown to be a ribosomal RNA m6A methyltransferase, validating our RNAi screen approach^1^. The second methyltransferase, C38D4.9, is an ortholog of the human METTL5 gene (Supplementary Fig. S2).

To verify the RNAi screen results, we utilized genetic mutant animals for ZCCHC4 (syb804) and METTL5 (tm4561) (Fig. 1a). Animals mutated for either methyltransferase displayed nearly 50% reduction in global m6A levels in total RNA (Fig. 1b). Moreover, the global m6A level in *mettl-5 zcchc-4* double mutant animals decreased to detection limit in total RNA (Fig. 1b). Given that ribosomal RNAs constitute the majority of cellular RNA and hence contribute to the majority of global m6A, we investigated whether the substantial reduction in m6A in these two methyltransferase mutants was due to a loss of ribosomal RNA m6A methylation. We isolated small and large ribosomal RNA subunits and determined m6A levels by HPLC–MS/MS. The z*cchc-4* animals lost m6A methylation on the large subunit rRNA (Fig. 1c). This methyltransferase has been reported to methylate 28S rRNA in human cells, suggesting that the rRNA methyltransferase function of ZCCHC4 is conserved in *C. elegans*^1^. While the large rRNA subunit m6A methylation is lost in the *zcchc-4-*mutant animals, the small rRNA subunit m6A methylation is not affected, indicating that m6A methylation events of large and small subunit rRNAs are independent of each other.

**Fig. 1:**
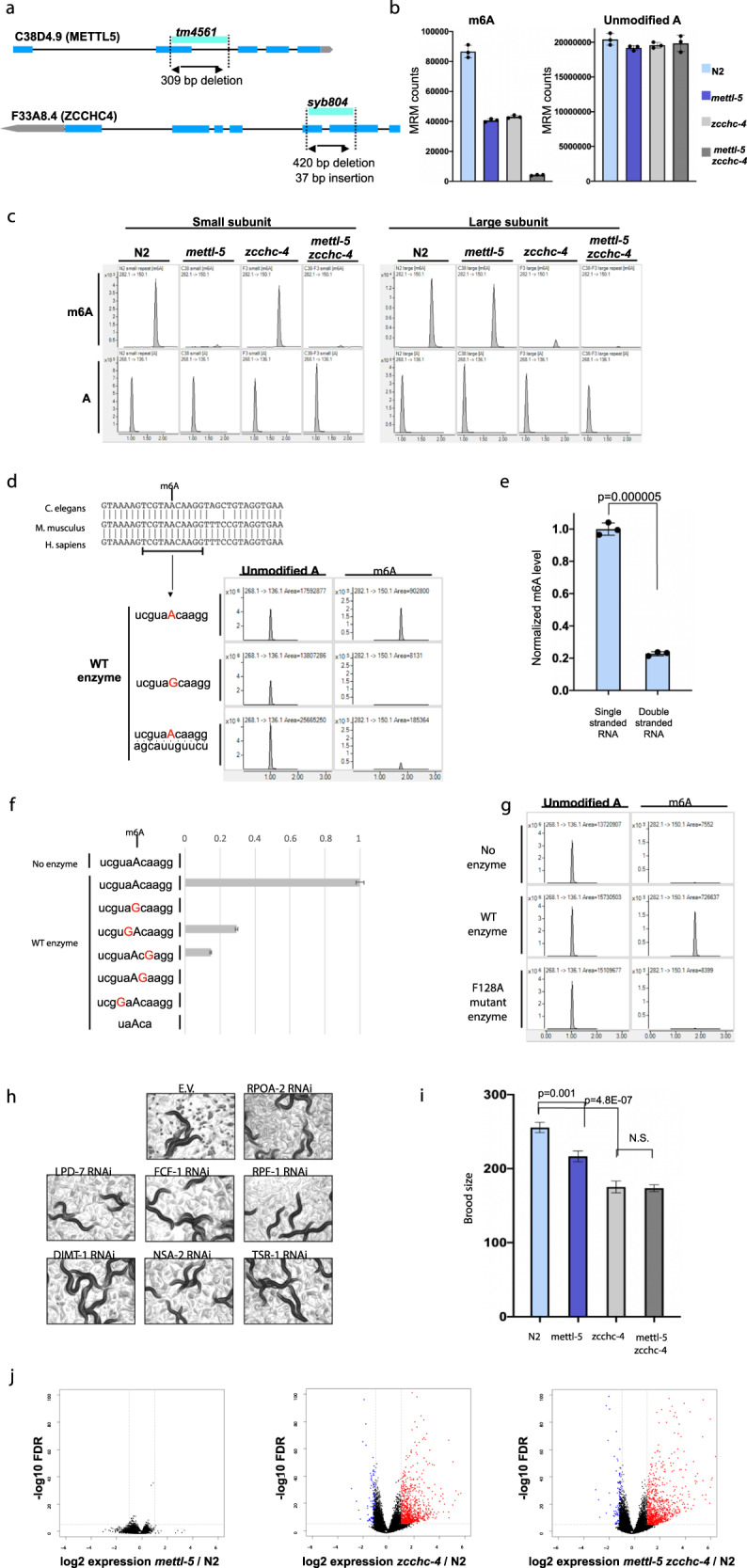
Landscape of RNA m6A methylation in *C.elegans*. **a** Diagrams depicting the *mettl-5* and *zcchc-4* mutants used in this study. **b** HPLC–MS/MS measurement of m6A and unmodified A MRM counts of total RNA from indicated worm strains. The experiments were performed in biological triplicates and error bars represent standard deviation. **c** HPLC–MS/MS peaks of small and large subunit ribosomal RNA isolated from indicated worm strains. **d** Worm, mouse, and human rRNA sequence alignment encompassing 11 bp RNA oligo sequence used in in vitro methylation reactions. HPLC–MS/MS peaks of in vitro methyltransferase reactions employing recombinant wild-type worm METTL5 protein and single or double-stranded RNA oligos with indicated sequences. **e** Quantification of HPLC–MS/MS analysis of in vitro methylation reactions using single stranded or double stranded 11 bp RNA oligos that are shown in **d**. Error bars represent standard deviation. **f** Quantification of HPLC–MS/MS analysis of in vitro methylation reactions employing recombinant worm METTL5 and RNA oligos with the indicated sequences, which demonstrates the substrate sequence and length specificity of METTL5 enzyme. **g** HPLC–MS/MS peaks of in vitro methylation reactions employing 11 bp rRNA oligo using wild-type or catalytically inactive F128A mutant recombinant METTL5 enzyme. **h** Images of worm plates showing adults and worm embryos indicating decrease in fertility after RNAi-mediated knock down of indicated rRNA biogenesis genes—rpoa-2 (F14B4.3), lpd-7 (R13A5.12), fcf-1 (F30A10.9), rpf-1 (F44G4.1), dimt-1 (E02H1.1), nsa-2 (W09C5.1), tsr-1 (F10G7.1) and E.V. (empty vector). **i** Bar graph depicting the brood size of indicated worm strains at 20 °C. Error bars represent standard error. **j** Volcano plots depicting statistically significant gene expression changes of embryos of indicated strains. Red depicts upregulated transcripts whereas blue downregulated transcripts.

Next, we investigated rRNA methylation levels in the *mettl-5* mutant. The loss of this methyltransferase resulted in a complete loss of m6A from the small subunit rRNA, suggesting that METTL5 is required for small subunit rRNA m6A methylation in *C. elegans*, as it was recently shown in mammalian cells^2^. Moreover, although m6A was lost in the small subunit rRNA, the large subunit m6A methylation in the *mettl-5* animals was not affected, further indicating that m6A methylation of small and large ribosomal subunits are independently mediated by two separate enzymes (Fig. 1c).

Ribosomal RNA transcription, processing, and modifications are highly regulated and coordinated^3^. Next, we asked whether the loss of METTL5 and hence the loss of small subunit m6A methylation affected other abundant ribosomal RNA modifications. While global m6A level decreased substantially in the *mettl-5* mutant, none of the other measured RNA modifications were significantly affected by the loss of this enzyme, suggesting that METTL5 is specific for m6A methylation (Supplementary Fig. S3a). We next investigated a potential crosstalk between m6A and other adenine modifications. Interestingly, the loss of nearly all global m6A methylation in the *mettl-5 zcchc-4* double mutant animals did not alter 2-O-methyladenosine (Am), 1-methyladenosine (m1A) or N6, N6-dimethyladenosine (m6,6A), suggesting that these other adenine modifications are not dependent on m6A. Moreover, there is no global crosstalk between global levels of m6A and the other adenine modifications (Supplementary Fig. S3b).

Next, we tested if METTL5 is sufficient to methylate ribosomal RNA in vitro. Ribosomal RNA is highly evolutionarily conserved among eukaryotes (Fig. 1d). We performed in vitro methylation assays with recombinant worm METTL5 using an 11 nucleotide RNA oligo that spans the m6A sequence (Fig. 1d). Recombinant METTL5 efficiently methylated this oligo and generated m6A (Fig. 1d). Adenosine at position 1717 in *C. elegans* corresponds to A1832 in human 18S rRNA, which carries m6A. Altering this adenosine to guanosine completely abolished the activity of METTL5 (Fig. 1f). Although there are three other adenosine residues on this altered RNA substrate, METTL5 did not show any methyltransferase activity for those sites, indicating the specificity of METTL5 for its target adenosine. Furthermore, mutating nearby residues on this RNA substrate either completely or substantially decreased METTL5 activity further indicating the specificity of this enzyme to its target rRNA motif (Fig. 1f). Moreover, a 5 nucleotide RNA oligo with the same methylation motif was not methylated by METTL5, whereas 11 nucleotide RNA was efficiently methylated, indicating that RNA length as well as RNA sequence are important for METTL5-mediated methylation (Fig. 1f).

Importantly, the activity of the enzyme diminishes significantly when its target sequence forms double-stranded RNA (Fig. 1d and e). In the mature ribosome, A1832 residue is in mostly double-stranded Helix 44^2^. This suggests that METTL5-mediated m6A methylation of rRNA takes place during rRNA processing before this target sequence becomes double stranded within Helix 44. Indeed, in human cells, METTL5 displays mostly nucleolar subcellular localization where early rRNA transcription and processing occur^4^, further suggesting that METTL5-mediated rRNA methylation takes place in nucleoli during ribosome biogenesis.

Given that loss of METTL5 results in a complete loss of m6A on the small rRNA subunit in vivo and recombinant METTL5 is sufficient for methylation of its target rRNA motif in vitro, we conclude that METTL5 is an rRNA m6A methyltransferase. Indeed, METTL5 contains the canonical m6A methyltransferase motif, NPPF (Supplementary Fig. S2). Mutation of the conserved active site residue F128 to alanine resulted in a complete loss of m6A methyltransferase activity in vitro (Fig. 1g), further establishing METTL5 as an RNA m6A methyltransferase, together with METTL3, METTL16, and ZCCHC4^5^.

Do METTL5 and ZCCHC4 rRNA methyltransferases methylate other types of RNAs such as messenger RNA in *C. elegans*? To address this question, we utilized mRNA-enriched total RNA samples from embryos and performed antibody-based global m6A mapping. We detected an m6A enrichment on the small rRNA subunit, which was lost in the *mettl-5-*mutant animals, confirming that METTL5 is the small rRNA subunit methyltransferase (Supplementary Fig. S4). Although m6A enrichment was observed by antibody-mediated m6A-RIP-Seq, this observed signal on coding transcripts was highly variable among replicates (Supplementary Fig. S5c). Mammalian mRNA displays a specific m6A enrichment over gene bodies, where high enrichment is observed near stop codons and 5′ UTRs, which is a result of the METTL3 complex activity^5^. The signal observed on worm transcripts does not show a clear enrichment pattern over the gene bodies by metagene analysis, indicating a lack of a regulated pattern of enzymatic activity (Supplementary Fig. S5a). Furthermore, upon manual inspection of the enriched regions, many fell into low-complexity or adenine-rich regions, suggesting background antibody signal. Moreover, no clear methylation motif was observed in RNA, indicating a lack of a methyltransferase target motif (Supplementary Fig. S5b). Importantly, apparent enrichment signals did not change in RNA from methyltransferase mutant embryos (Supplementary Fig. S5c). In addition, purified mRNA samples failed to display significant m6A signal by HPLC–MS/MS. Although we cannot rule out the possibility of a low-level mRNA methylation in specific tissues or developmental stages under different conditions that were not tested in this study, we propose that *C. elegans* mRNA lacks m6A modification, which is abundant and regulated in mammalian cells. This is consistent with the fact that the worm genome does not contain genes that encode the mRNA m6A methylation machinery homologs, such as the methyltransferases METTL3/METTL14 and demethylases FTO and ALKBH5 or YTH domain m6A mRNA reader proteins. On the other hand, the *C. elegans* genome does contain genes encoding two other known evolutionarily conserved m6A methyltransferases, METTL16 (mett-10) and METTL4 (C18A3.1). It has recently been reported that in multiple organisms, these two methyltransferases methylate N6 positions of adenines in U6 and U2 snRNAs, respectively^6–9^. In summary, *C. elegans* ZCCHC4 and METTL5 are primarily rRNA methyltransferases.

The fact that ZCCHC4 and METTL5 are both rRNA methyltransferases, which are expressed throughout the organism^10^, raises the possibility that they may affect ribosome biogenesis and/or function and *C. elegans* development. To address this question, we first attempted to identify the physiological impact of disruption of ribosome biogenesis in general. We took advantage of efficient RNAi knock down in *C. elegans*. Knock down of a core component of the RNA Polymerase 1 POLR1B ortholog RPOA-2 resulted in severe loss of fertility (Fig. 1h). To test if this effect on fertility was common to disruption of ribosome biogenesis in general, we knocked down other conserved proteins in this pathway. Knock down of ribosome biogenesis proteins that are involved in both small and large subunit biogenesis resulted in decreased fertility (Fig. 1h), suggesting that this pathway is important for germline homeostasis and disruption of this pathway results in decreased fertility in *C. elegans*.

To test whether loss of the large and small rRNA subunit methyltransferases and hence loss of m6A affects fertility, we measured brood sizes in *mettl-5* and *zcchc-4* animals. As expected, the loss of either methyltransferase resulted in a significant decrease in brood size, indicating that these enzymes are required for germline homeostasis in *C. elegans* (Fig. 1i).

Many proteins involved in ribosome biogenesis do not have catalytic activity. To test whether enzyme activity of METTL5 is physiologically important, we utilized animals that have catalytically inactive METTL5. Point mutation in the conserved SAM-binding motif (G55E) resulted in loss of activity in vitro (Supplementary Fig. S6a and b). As expected, global m6A level decreased significantly in vivo (Supplementary Fig. S6c). More importantly, the loss of METTL5 catalytic activity also resulted in a significant decrease in brood size, phenocopying the *mettl-5* knockout animals, indicating that catalytic activity of METTL5 and hence the m6A modification on small subunit rRNA is important for brood size in *C. elegans* (Supplementary Fig. S6d).

Next, we determined transcriptomic changes in embryos in response to the loss of these methyltransferases. Compared to wildtype N2 embryos, METTL5-deficient embryos displayed minimal transcript changes (Fig. 1j). In contrast, the loss of ZCCHC4 resulted in major transcript mis-regulation (Fig. 1j). 764 genes were upregulated, whereas 127 genes were downregulated due to loss of ZCCHC4 (Supplementary Fig. S7, Table S1). The transcript changes parallel the phenotypes the mutant worms display, i.e., the loss of ZCCHC4 results in a higher level of transcript mis-regulation with more reduction in brood size compared to the *mettl-5* mutant animals with minor impact on transcription and loss in fertility.

Previous studies reported that conditions that inhibit translation extend lifespan in *C. elegans*^11^. Given that both ZCCHC4 and METTL5 have been implicated in controlling protein synthesis, we performed longevity assays with both mutant animals in order to gain additional insights into the physiological impact of m6A. *zcchc-4* mutant animals have extended lifespan whereas *mettl-5* mutant animals do not display significant changes in lifespan (Supplementary Fig. S8). Moreover, the *mettl-5;zcchc-4* double mutant animals are similar to *zcchc-4* mutant animals in terms of changes in gene expression, brood size, and lifespan (Fig. 1j, i, Supplementary Figs. S7, S8), suggesting that these phenotypic changes are mostly due to the loss of ZCCHC4 rather than METTL5.

In summary, we analyzed RNA m6A methylation in *C. elegans* and characterized the two major methyltransferases that contribute to almost all of m6A RNA methylation in *C. elegans*. We further characterized an evolutionarily conserved RNA m6A methyltransferase that is involved in ribosome biogenesis. Our results indicate that METTL5 methylates the small subunit of ribosomal RNA during ribosome biogenesis. We further characterized the physiological importance of both the small and large subunit m6A methyltransferases and showed that these enzymes are important for fertility and longevity in *C. elegans*. Given that there are no orthologs of known m6A demethylases ALKBH5 or FTO in *C. elegans*, we predict that RNA m6A methylation is not a reversible, dynamic modification in this organism. The fact that both small and large ribosomal m6A sites are fully methylated in some human cells is further consistent with this idea^12^.

We conclude that *C. elegans* utilizes m6A methylation as higher eukaryotes do, but almost all RNA m6A methylation in this organism is on ribosomal RNAs and is mediated by METTL5 and ZCCHC4. The two additional RNA methyltransferases, METTL4 and METTL16, contribute to U2 and U6 snRNA methylation, respectively. There are no other candidate methyltransferases with the known N6-Adenine methylation catalytic motif, (D/N)-P-P-(F/W), in the *C. elegans* genome, although we cannot rule out the presence of novel methyltransferases with different catalytic mechanisms that might contribute to minor levels of m6A methylation not detectable by our current analysis. These findings establish *C. elegans* as a model organism to investigate m6A methylation in ribosome biogenesis, translation, and RNA splicing via these four m6A methyltransferases (Supplementary Fig. S9).

*Note-added-in-proof*: While this manuscript was under review, multiple studies showed that METTL5 mediates m6A methylation on the small subunit ribosomal RNA in flies, worms, mice, and human cells^13–16^. Collectively, our findings as well as the above referenced recent studies further support the conclusion that METTL5 is an evolutionarily conserved rRNA m6A methyltransferase.

## Supplementary information

Supplementary Information

## Supplementary information

**Supplementary information** accompanies the paper at (10.1038/s41421-020-00186-6).
