# Supplementary material for: Analysis of m6A RNA methylation in Caenorhabditis elegans
Source: Cell Discov. 2020 Jul 14;6:47. doi: 10.1038/s41421-020-00186-6 (PMC7359367; doi:10.1038/s41421-020-00186-6)

Methods:

RNA purification

Total RNA was isolated from animals with TRIzol reagent following manufacturer's instructions. Briefly, L4/young adult worms were washed off plates and sedimented by gravity, washed twice more to remove potential bacterial contamination. The worm pellet was resuspended in 500 uL TRIzol, which was flash frozen in liquid N_2 and thawed five times to lyse the cells. 100 uL chloroform was added and mixed by vortexing. The RNA was isolated by isopropanol precipitation of the aqueous phase after centrifugation at 4^oC.

For ribosomal RNA purification, 10 ug total RNA was run on 1% agarose gel for an hour to resolve large and small subunit rRNA bands. The corresponding RNA bands were cut from the gel and purified with Zymoclean™ Gel RNA Recovery Kit from Zymo Research.

HPLC-MS/MS analysis of RNA

250 ng to 500 ng RNA was digested with 0.5U P1 nuclease (Sigma-Aldrich) or 100U S1 nuclease (NEB) at 37^oC for 2 hours and dephosphorylated with 1U rSAP (NEB) at 37^oC for 1 hour. The 100 µL samples were filtered with Millex-GV 0.22u filters.

For detection of m6Am, 250 or 500 ng of mRNA was de-capped with 0.5U of Cap-Clip enzyme (Cellscript) in 20 uL reaction volume for an hour at 37^oC prior to P1 nuclease digestion. m6Am modification was not detected in C. elegans mRNA or total RNA.

10 uL from each sample is injected into Agilent 6470 Triple Quad LC/MS instrument. The samples were run in mobile phase buffer A (water with 0.1% Formic Acid) and 2 to 20%

gradient of buffer B (Methanol with 0.1% Formic Acid). MRM transitions were measured for adenosine (268.1 to 136.1), guanosine (284.1 to 152.1), 2'-O-methyladenosine (Am) (282.1 to 136.1), N6-methyladenosine (m6A) (282.1 to 150.1), N6,2-O-dimethyladenosine (m6Am) (296.1 to 150.1), N6,N6-dimethyladenosine (m6,6A) (296.1 to 164.1), 7-methylguanosine (m7G) (298.1 to 166.1), 1-methylguanosine (m1G) (298.1 to 166.1), 2-methylguanosine (m2G) (298.1 to 166.1). For LC/MS-MS data collection and analysis, Agilent Mass Hunter LC/MS Data Acquisition Version B.08.00 and Quantitative Analysis Version B.07.01 softwares were used.

Worm Strains, culture and genotyping

mettl5 (C38D4.9) deletion strain tm4561 was acquired from Mitani Lab (Tokyo Women's Medical University). *mettl5* (G55E) (gk173456 allele, VC20285 strain) is acquired from CGC, which is funded by NIH Office of Research Infrastructure Programs (P40 OD010440). *zcchc4* (F33A8.4) deletion strain (syb804) was acquired from SunyBiotech. All the strains were backcrossed to N2 multiple times.

C38D4.9 tm4561 genotyping forward	TGATGGAAATGATCGATGAAA
C38D4.9 tm4561 genotyping reverse	CGCATCTGGAAGAATTGAAAG

These primers will amplify a fragment of 451 basepairs from wild type, 142 bp from tm4561.

F33A4.9 genotyping forward	TAAAGGCGCACATCCGTTTT
F33A4.9 genotyping reverse	CAAGTTCTTCCAACTGATGACCT

Wild type would give 600 bp product. syb804(-420bp+37bp) KO would give 217 bp

C38D4.9 G55E (gk173456) genotyping forward	GCCGCGCTTCAAAATTTTATT
C38D4.9 G55E (gk173456) genotyping reverse	ACTTCAGCTGTTTCCAAATTTCT

Wild-type is cut by Hpy188I (178/177). Mutant is cut by BtsCI (190/188)

RNAi knock down was carried out by cloning corresponding gene fragments into plasmid pL4440. HT115 E. coli strain was used for RNAi knock downs on NGM plates with IPTG. The worms were kept on RNAi bacteria for 2 generations.

To determine brood size, animals were age-synchronized by hypochlorite treatment and grown on plates seeded with *Escherichia coli* OP50. Ten L3/L4 animals were manually transferred to individual plates seeded with OP50. Adults were daily transferred to new plates for 3 days and the number of L3 larvae laid on each plate was counted.

m6A RIP-Seq

mRNA was obtained from total RNA (100 ug) by purifying twice with a rRNA depletion kit (RiboMinus Eukaryote Kit v2) following manufacturer's instructions.

m6A RIP-seq experiments were performed as previously described (Sendinc, David-Valle et.al, Molecular Cell, 2019). Briefly: 5 ug of mRNA are fragmented at 98ºC for 3 min in 200 ul of fragmenting buffer (10 mM Tris pH 7.4, 10 mM $ZnCl_2$) and quenched with 70mM EDTA. 50 uL of Protein G Dynabeads (Invitrogen) were incubated with 250 ug of rabbit monoclonal anti-m6A (Cell Signaling Technologies, Cat #56593S) at 4ºC for 30 mins. The beads/antibody slurry was added to the fragmented mRNA and incubated in rotation for 1 h at 4ºC. Beads were washed twice with 200 uL of reaction buffer (150 mM NaCl, 10 mM Tris-HCl, 0.1% Triton X-100, pH 7.5), twice with 200 uL of low salt buffer (50 mM NaCl, 10 mM Tris-HCl, 0.1% Triton X-100, pH 7.5) and twice with 200 uL of high salt buffer (500 mM NaCl, 10 mM Tris-HCl, 0.1% Triton X-100, pH 7.5). Immunoprecipitated RNA was eluted in TRIzol and purified with the RNA clean and concentrator Kit -5 (Zymo, Cat #R1013). Illumina sequencing libraries were prepared with the NEBNext Ultra II Directional Library Prep Kit for Illumina from NEB (#E7760), following manufacturer's instructions. RNA-Seq libraries were generated from three biological replicates

for Input (RNA-Seq) and m6A-immunoprecipitated mRNA. Libraries were sequenced in a NextSeq 550 with an average coverage of 30 million reads per sample.

Bioinformatic analyses

Reads were aligned with hisat2 (v2.0.4, parameters: --no-unal --rna-strandness R) using the gtf annotation WBcel235 downloaded from Ensembl genes v96 as reference. Sam files were converted to bam files using samtools (v 1.9, parameters: -bSq 10). Bigwig files were generated using the bamCoverage program from the deepTools package (v3.3.0, parameters: -bs 20 --normalizeUsing BPM --skipNAs --ignoreDuplicates).

Gene counts for the Input files (RNAseq experiments) were obtained with the featureCounts program fron the Rsubread package (v 1.34.1, parameters minMQS=10, allowMultiOverlap=F, largestOverlap=T, strandSpecific=2) using the WBcel235 annotation from Ensembl genes v96. Normalization and dispersion factors for RNAseq analysis were calculated using the functions calcNormFactors() and estimateDisp() from the edgeR package (v 3.26.1). DEG were calculated using the glmFit() and glmLRT() functions from edgeR. Genes with a |logFC| > 1 and FDR < 1e-5 were considered differentially expressed.

m6A peaks were calculated with the R package exomePeaks (v 2.17) using a FDR cutoff of 1e-10 and a fold enrichment of 10. Only consistent peaks (identified in all replicates) were included. Peaks within genes with low expression level (cpm < 1) were ignored. 805 m6A peaks (Supplementary Table 2) within 648 genes were identified.
Heatmaps of the log-fold m6A/Input enrichment were generated with the computeMatrix (parameters: scale-regions -m 100 -bs 5 --metagene) and plotHeatmap programs from the deepTools suite. Metagene plots were obtained with computeMatrix (parameters: scale-regions -m 500 -bs 10 --metagene) and plotProfile from the same suite.

Fasta sequences from the peaks identified by exomePeak were obtained with the fastaFromBed program from the bedtools suite (v2.29.2, parameters: -s -name -split). Motif analysis was performed with the MEME-ChIP tool (v5.1.1) using default parameters and the Uniprobe Worm (Cell09) motif database.

The sequencing data in this study can be accessed via GEO number GSE148786.

Lifespan Analysis

Lifespan assays were conducted at 20°C on OP50. Strains were grown under optimal conditions for at least two generations before washing off mixed-staged populations to obtain synchronized embryos, marking Day 1 of the lifespan assay. When embryos reached adulthood, adults were transferred onto fresh OP50 plates at least every other day until the end of the reproductive period. Animals that died bagged, burst or crawled off the plate were censored from the analysis. Statistical significance was assessed using the log-rank test (Mantel-Cox test).

Recombinant Protein Purification

Full-length *C. elegans* METTL5 gene was cloned into pGEX-4T1 vector. It was expressed overnight in 500 mL Rosetta bacteria (Novagen) culture induced with 0.1 mM IPTG at 18°C. The cells were lysed in 15 mL cold lysis buffer (50 mM Tris pH 7.4, 150 mM NaCl, 0.05% NP-40, 1mM PMSF) with 0.25 mg/mL chicken lysozyme on ice for 30 min. The cell lysate was sonicated on ice and cleared with centrifugation for 20 min at 10000 rpm at 4°C. The GST tagged recombinant protein was bound to Glutathione Sepharose 4B beads (GE Healthcare) at 4°C with rotation for 3 hours. The beads were washed with lysis buffer containing 500 mM NaCl. The untagged recombinant protein was eluted off the beads with overnight rotation at room

temperature with 5U of thrombin in thrombin cleavage buffer (50 mM Tris pH 8.0, 150 mM NaCl, 5mM $CaCl_2$). The protein preparation was supplemented with 10% glycerol and stored at -80^oC.

In vitro RNA Methylation Assays

The in vitro enzyme assays were carried out by incubating 1ug full-length untagged recombinant METTL5 enzyme with corresponding RNA oligos with varying lengths and designated mutations. Single stranded RNA oligos are acquired from IDT. The reactions were performed at 37^oC in 50 mM Tris-HCl (pH 7.5 at 25 °C), 5 mM β-ME, 10 mM EDTA and 80 μM S-adenosylmethionine (SAM) buffer for 1 hour. The reactions were further processed as described in HPLC-MS/MS analysis of RNA methods section. The levels of the final product (m6A) formed was determined using mass spectrometry by monitoring the amount of m6A in each aliquot.

Supplementary Figure Legends:

Supplementary Figure 1 Candidate RNAi screen for identification of m6A methyltransferases. HPLC-MS/MS MRM counts of normalized m6A after RNAi knock down of candidate methyltransferases

Supplementary Figure 2 *C. elegans* C38D4.9 and F33A8.4 are orthologs of human METTL5 and ZCCHC4. Alignments of protein sequences of human and worm METTL5 and ZCCHC4 proteins using Protein BLAST. The methyltransferase catalytic motifs are highlighted.

Supplementary Figure 3 m6A is independent of other RNA methylations. a Normalized HPLC-MS/MS MRM counts of indicated RNA modifications of total RNA from N2 and *mettl-5* mutant worms. **b** Normalized HPLC-MS/MS MRM counts of indicated RNA modifications from N2, *mettl-5*, *zcchc-4* and *mettl-5;zcchc-4* double mutant worms

Supplementary Figure 4 Loss of METTL5 results in loss of m6A enrichment of small subunit rRNA. Genome browser view of input RNA and m6A IP reads on small subunit rRNA from RNA samples of 2 replicates from N2 and *mettl-5* mutant embryos. Scale for all the tracks: 0-4,500 FPKM. Small subunit rRNA m6A location is highlighted.

Supplementary Figure 5 Analysis of m6A enrichment of *C. elegans* transcripts.
a Metagene analysis at genes with consistent m6A peaks (see methods for details). log2 m6A enrichment over Input from three biological replicates of N2 embryos is shown. **b** Motif analysis of m6A enrichment with indicated p-values. Most of the enrichment observed is over low complexity sequences with highly repeated adenosines. **c** Heatmap displaying log2 m6A enrichment over Input of transcripts (n=648) from N2, *mettl-5*, *zcchc-4* and *mettl-5;zcchc-4* mutant embryos. There is no consistent significant change of m6A across the replicates.

Moreover, the observed m6A enrichment is over low complexity regions with highly repeated adenosines. Taken together, these demonstrate lack of significant m6A on coding transcripts.

Supplementary Figure 6 Catalytic activity of METTL5 is important for brood size

a Alignment of protein sequences of human and worm METTL5 using Protein BLAST. The methyltransferase catalytic motifs (NPPF) and the SAM binding motif (GxGxG) are highlighted. **b** HPLC-MS/MS peaks of *in vitro* methylation reactions employing 11 bp rRNA oligo using wild-type or SAM binding motif mutant (G55E) recombinant METTL5 enzyme. **c** HPLC-MS/MS measurement of m6A/A percentage of total RNA from N2 and *mettl-5* G55E mutant worm strains. **d** Bar graph depicting the brood size of indicated worm strains at 20°C

Supplementary Figure 7 Overlap of changes in transcripts of rRNA methyltransferase mutants. Venn diagrams showing overlaps between transcripts that are upregulated or downregulated in *zcchc-4* or *mettl-5;zcchc-4* double mutant embryos compared to N2 embryos, indicating significant transcriptional overlap between these two mutant strains

Supplementary Figure 8 Lifespan analysis of methyltransferase mutants. Plots indicating life span assays of **a** *mettl-5* **b** *zcchc-4* **c** *mettl-5;zcchc-4* mutant animals conducted at 20°C on OP-50. Statistical significance was assessed using the log-rank test (Mantel-Cox test).

Supplementary Figure 9 Landscape of RNA m6A methylation of *C. elegans*. Diagram depicting landscape of m6A RNA methylation in *C. elegans*. Majority of global m6A is contributed by ZCCHC4 and METTL5 enzymes which methylate large and small subunit rRNA during ribosome biogenesis, respectively. METTL4 and METTL16 enzymes contribute to minor level of global m6A by methylating U2 and U6 snRNA, respectively. The perturbation of m6A enzymes has effects on lifespan and fertility in *C. elegans*.

Supplementary Fig. S1 Candidate RNAi screen for identification of m6A methyltransferases

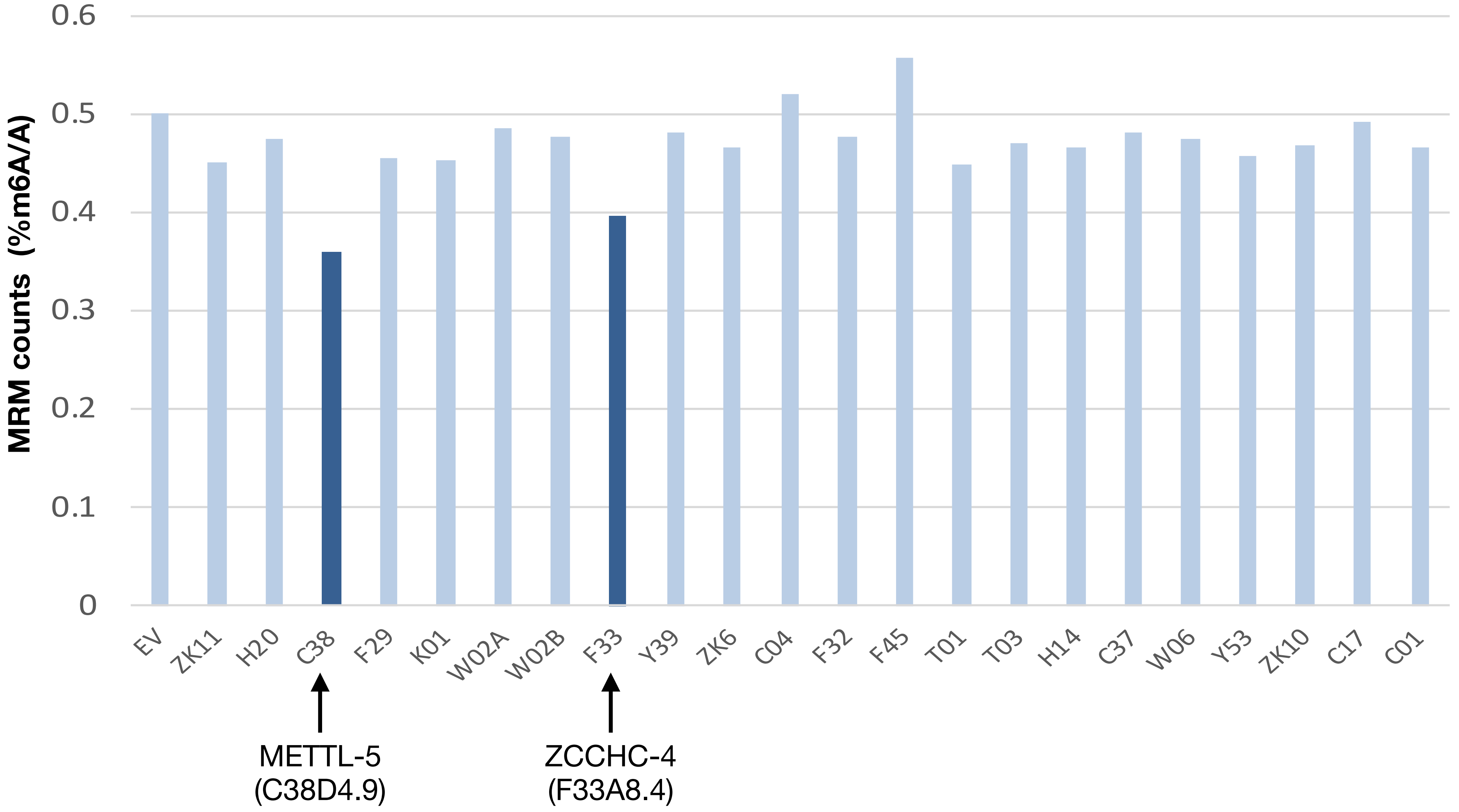

Supplementary Fig. S2 *C. elegans* C38D4.9 and F33A8.4 are orthologs of human METTL5 and ZCCHC4

METTL5

Score	Expect	Method	Identities	Positives	Gaps
169 bits(428)	8e-58	Compositional matrix adjust.	89/201(44%)	132/201(65%)	7/201(3%)

Methyltransferase motif (NPPF)

```
H. sapiens  13   LQQVDGFEKPKLLLEQYPTRPHIAACMLYTIHNTYDDIENKVVADLGCGCGVL-SIGTAM  71
                 L +++GFEKPK+ LEQY T    +A  M+   I   T     E K + D+GCGCG+L +     M
C. elegans  10   LNELEGFEKPKIKLEQYATSSELAVSMMEMIDETIG-FEGKKLIDIGCGCGMLMTTAATM  68

H. sapiens  72   LGAGLCVGFDIDEDALEIFNRNAEEFELTN-IDMVQCDVCLLSNRMSK-SFDTVIMNPPF  129
                       +G DID++AL+I +RN E   E+ +   +++Q D+     + + + +FD   ++NPPF
C. elegans  69   YELETVLGVDIDDEALKICSRNLETAEVQDRCELLQADILDPESDLPRGTFDVAVINPPF  128

H. sapiens  130  GTKNNKGTDMAFLKTALEMART--AVYSLHKSSTREHVQKKAAEWK-IKIDIIAELRYDL  186
                 GTKNN G DM F++   L+M R    +V+SLHKSSTR+++ K A +W   +   +   AE+R+ L
C. elegans  129  GTKNNAGIDMQFVQIGLQMVRPGGSVFSLHKSSTRDYILKNAKKWDGVGAECCAEMRWQL  188

H. sapiens  187  PASYKFHKKKSVDIEVDLIRF  207
                 PA+YKFHK+K+VDI VDLI F
C. elegans  189  PATYKFHKQKAVDIAVDLIHF  209
```

ZCCHC4

Score	Expect	Method	Identities	Positives	Gaps
190 bits(483)	4e-59	Compositional matrix adjust.	125/417(30%)	199/417(47%)	37/417(8%)

Methyltransferase motif (DPPF)

```
H. sapiens  24   GMEVVLPLDPAVPAPLCPHGPTLLFVKVTQGKEETRRFYACSACRDRKDCNFFQWEDEKL  83
                 G   VLP D      P C HGP LLF K    G E     F+AC+   R++ +     F+     K
C. elegans  67   GFFQVLPTDDLPQVPQCSHGPCLLFEK-KNGPEVEETFFACAIYRNQAEFCDFKLTFNKE  125

H. sapiens  84   SGARLAAREAHNRRCQPPLSRTQCVERYLKFIEL-----PLTQRKFCQTCQQLLLPDDWG  138
                 +G                    + + +  Y + +        P      +C+TC   +
C. elegans  126  TGEIEVKEAVEGEEDAEKKKKKKKIFGYNRIPKALTEMSPTDTVLYCKTCINVF------  179

H. sapiens  139  QHSEHQ-VLGNVSITQLRRPSQLLYPLENKKTNAQYLFADRSCQFLVDLLSALGFRRVLC  197
                   ++H+ V    V     L RP+ LL P+   +    +Q+ F+   +    +    +          +LC
C. elegans  180  -PNKHECVCEPVEREALERPTHLLPPVNEQHGESQFFFSTETLDVITKAVEKSKVDGILC  238

H. sapiens  198  VGTPRLHELIKLTASGDKKSNIKSLLLDIDFRYSQFYMEDSFCHYNMFNHHFFDGKTALE  257
                 +G PR+ E I+    +    + N+    LLD D R+++F+      +   Y+M    HFFD      +
C. elegans  239  IGAPRIFENIR---ALHPEKNV--FLLDYDKRFAKFFPSKQYAQYSMLVDHFFDKIAEPK  293

H. sapiens  258  VCRAFLQEDKGEGIIMVTDPPFGGLVEPLAITFKKLIAMWKEGQSQDDSHKELPIFW---  314
                 +    F    DK + I+M+TDPPFG   +EPL   + +K+    +             + KE   +F+
C. elegans  294  LMEFF---DKSKSILMITDPPFGVFMEPLLKSIEKMKKRFV------STGKEETLFYSMI  344

H. sapiens  315  IFPYFFESRICQFFPSFQMLDYQVDYDNHALYKHGKTGRKQSPVRIFTNIPPNKIILPTE  374
                 + P +     +        +F  M DY+V Y+ H LY+H +      ++ VR+FT++P    I L
C. elegans  345  VLPIYIRKYVLH--GNFWMSDYRVTYEGHKLYQHSE----KTIVRLFTDLPVECIDLKNV  398

H. sapiens  375  EGYRFCSPCQRYVSLENQHCELCNSCTSKDGRKWNHCFLCKKCVKPSWIHCSICNHC  431
                  GY+FC   C RYV+   N HC+ C +CTS +   KWNHC   C KCVKP ++HC+ C   C
C. elegans  399  AGYKFCEVCDRYVTERNVHCDRCQACTSVEQGKWNHCEKCDKCVKPRYVHCAQCARC  455
```

Supplementary Fig. S3 m6A is independent of other RNA methylations

a

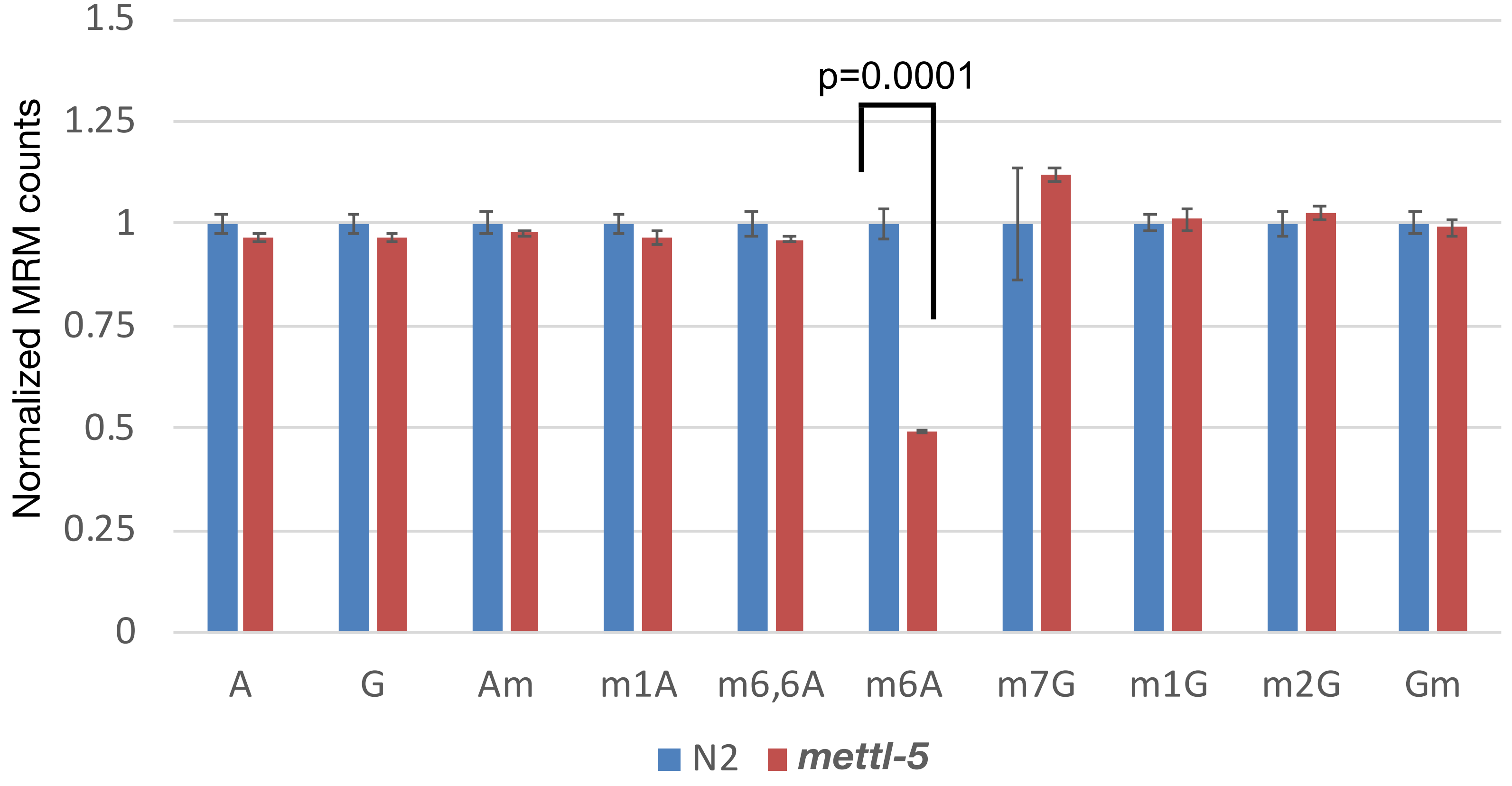

b

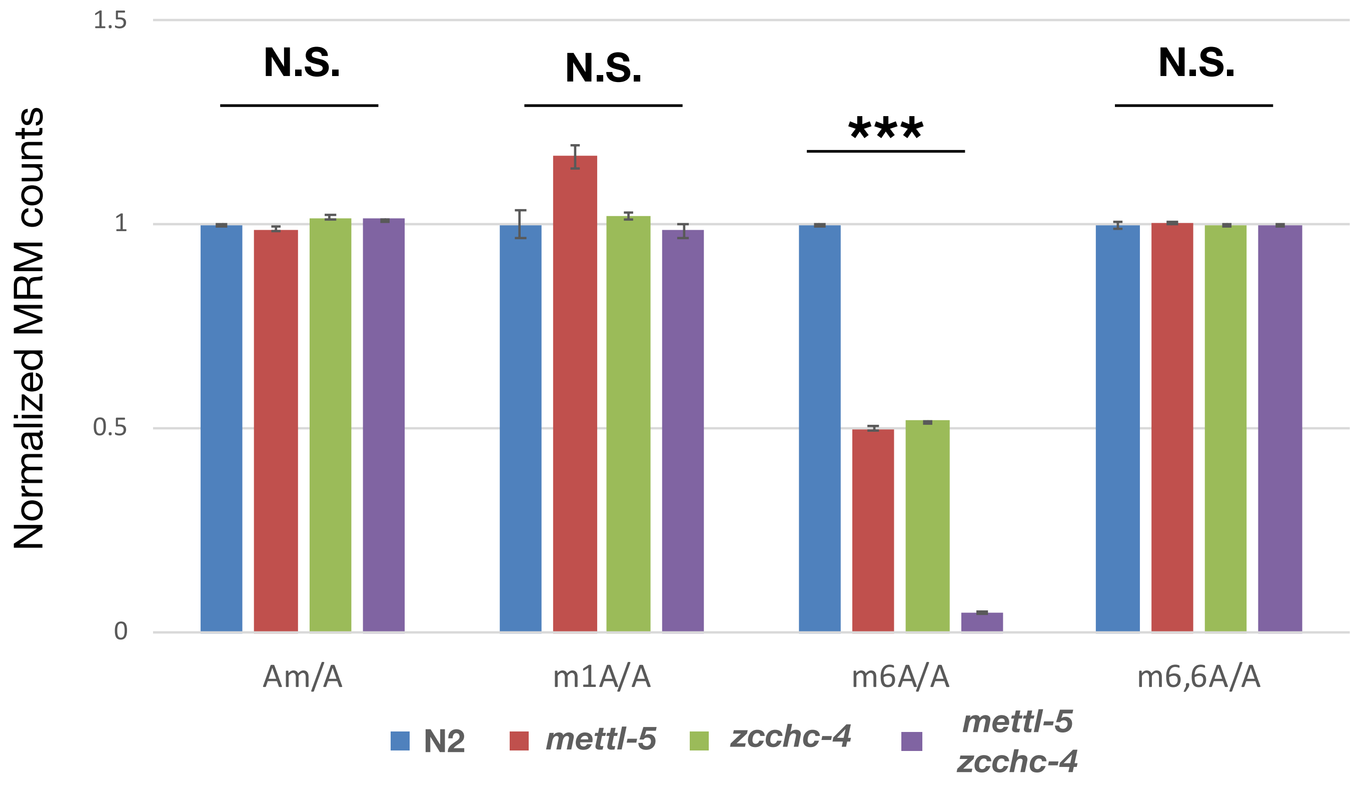

Supplementary Fig. S4 Loss of METTL5 results in loss of m6A enrichment of small subunit rRNA

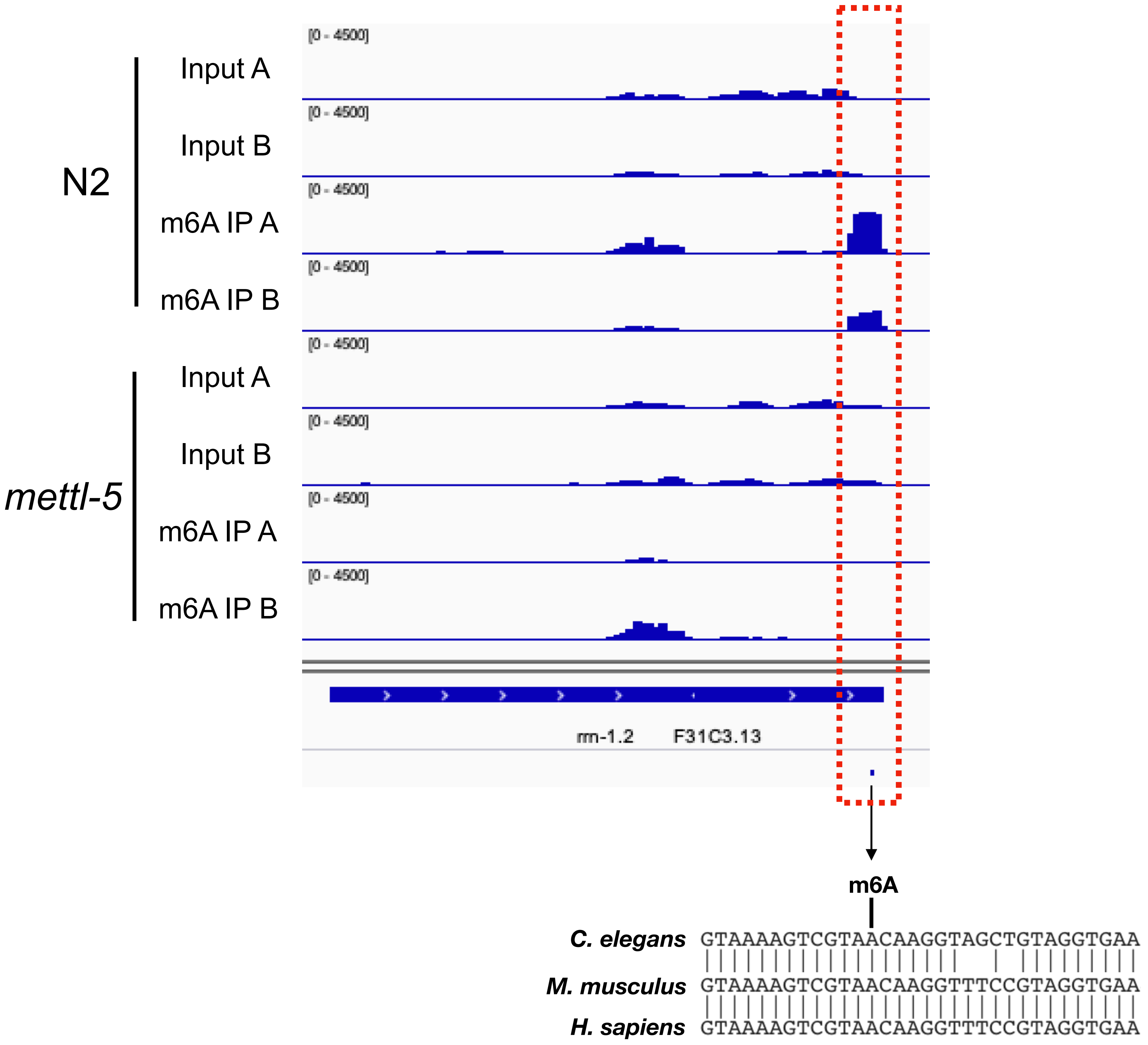

Supplementary Fig. S5 Analysis of m6A enrichment of *C. elegans* transcripts

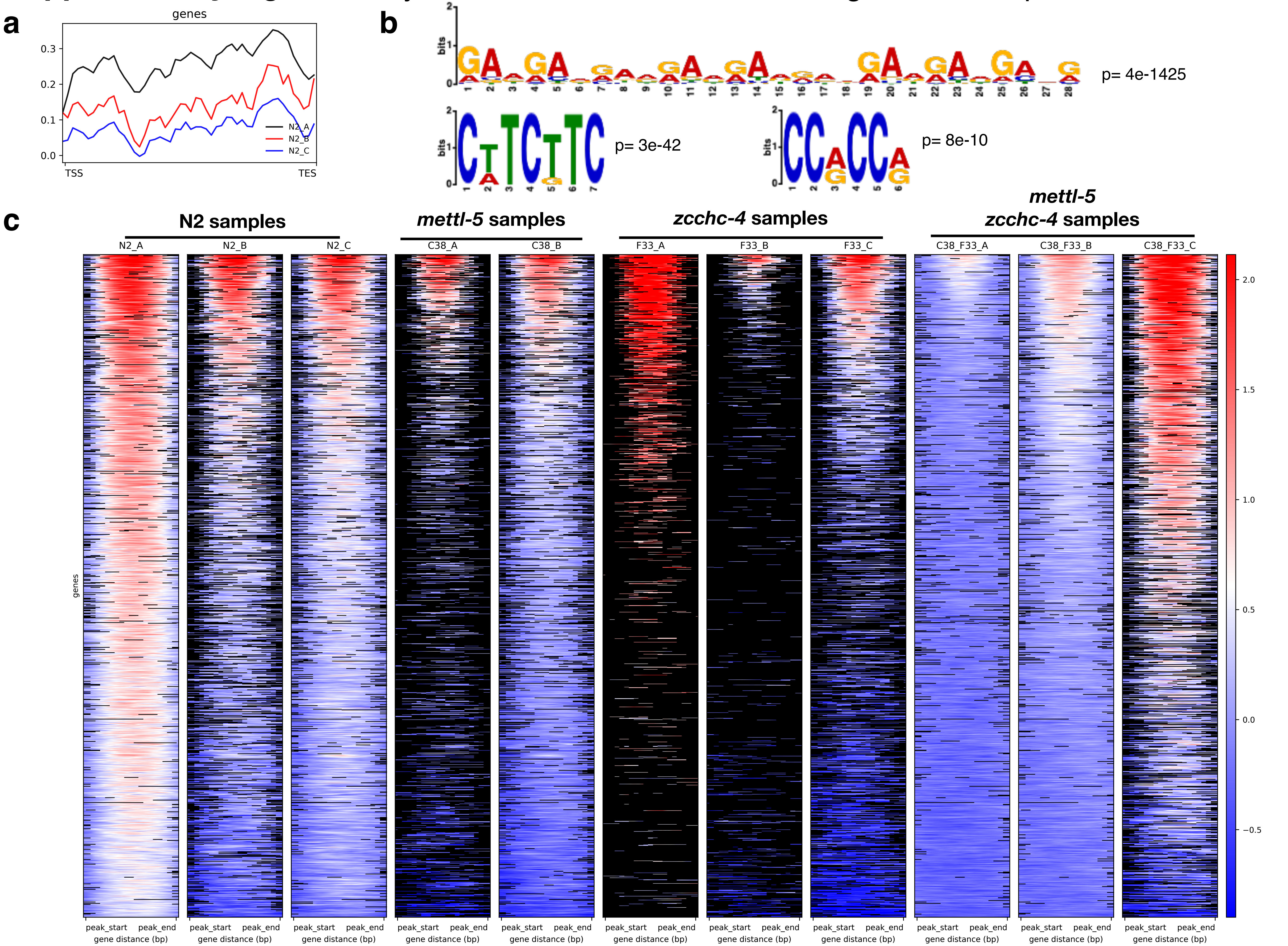

Supplementary Fig. S6 Catalytic activity of METTL5 is important for brood size

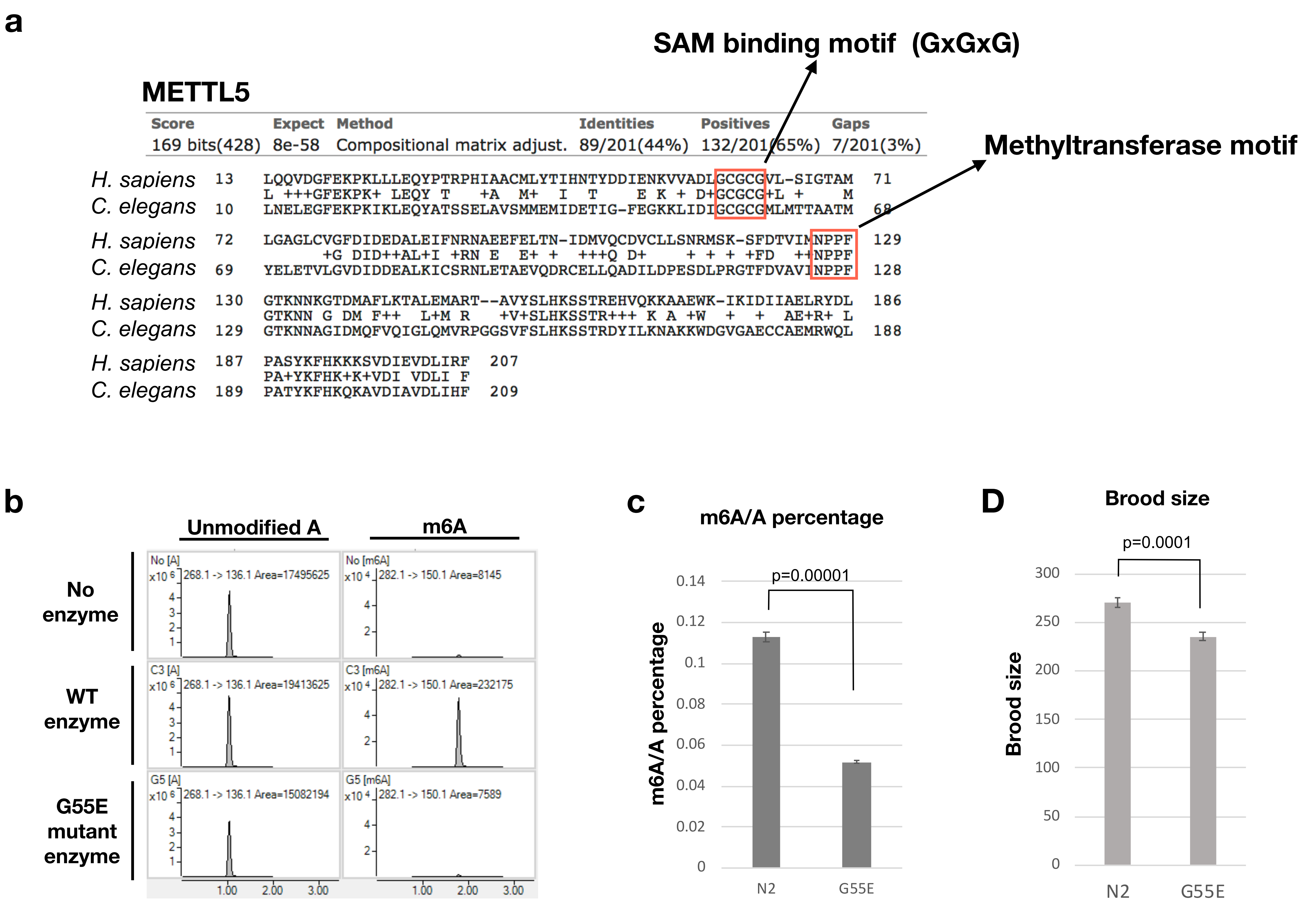

Supplementary Fig. S7 Overlap of changes in transcripts of rRNA methyltransferase mutants

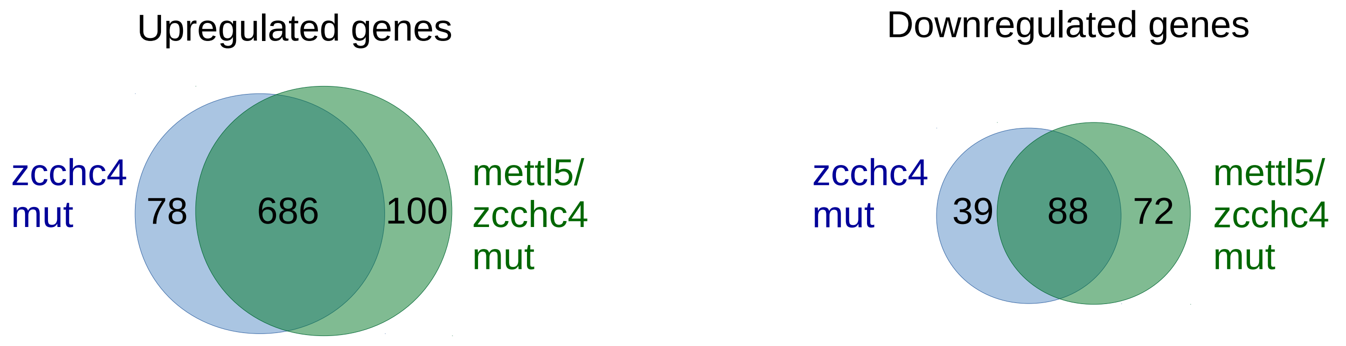

Supplementary Fig. S8 Lifespan analysis of methyltransferase mutants

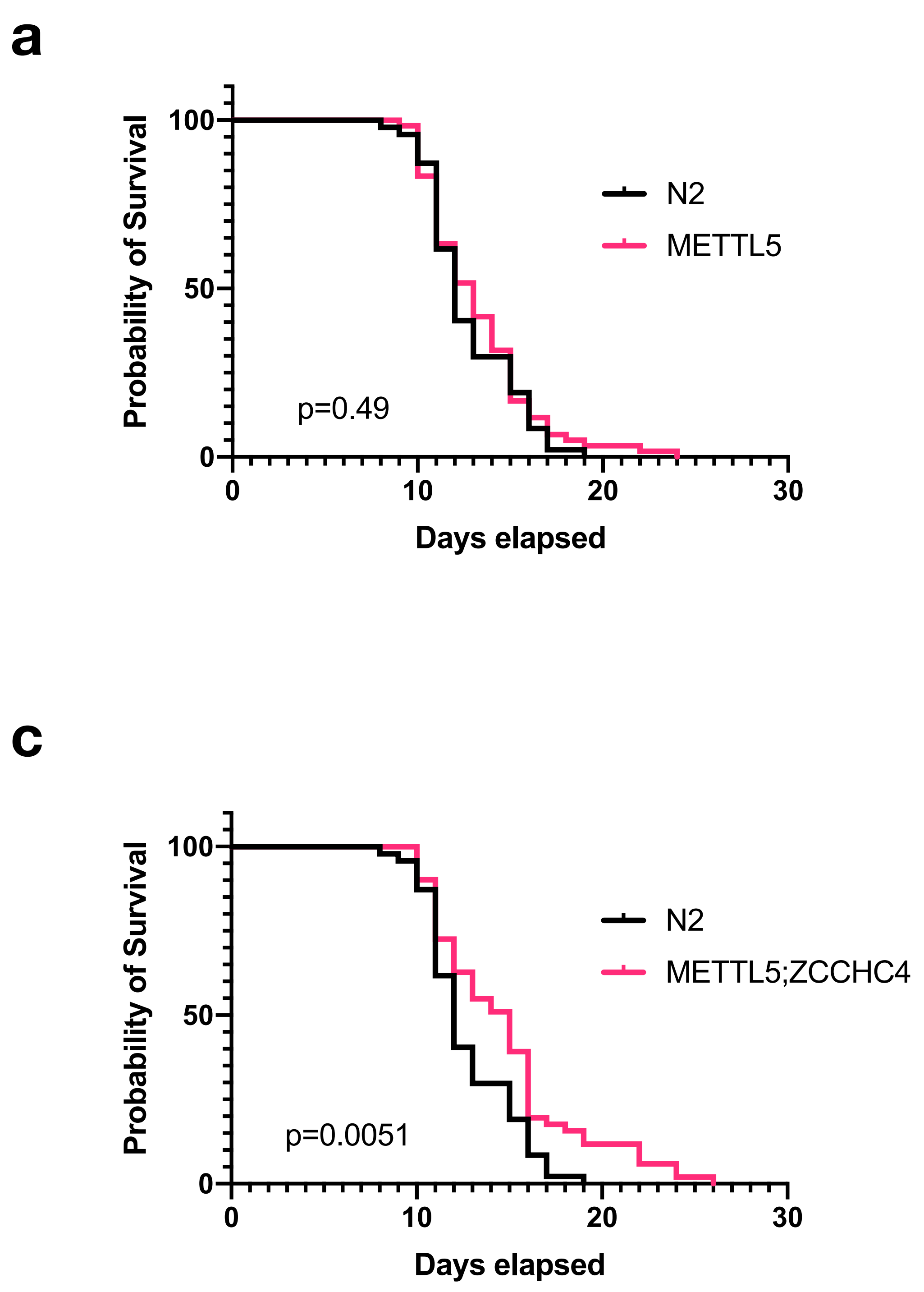

Supplementary Fig. S9 Landscape of RNA m6A methylation of *C. elegans*

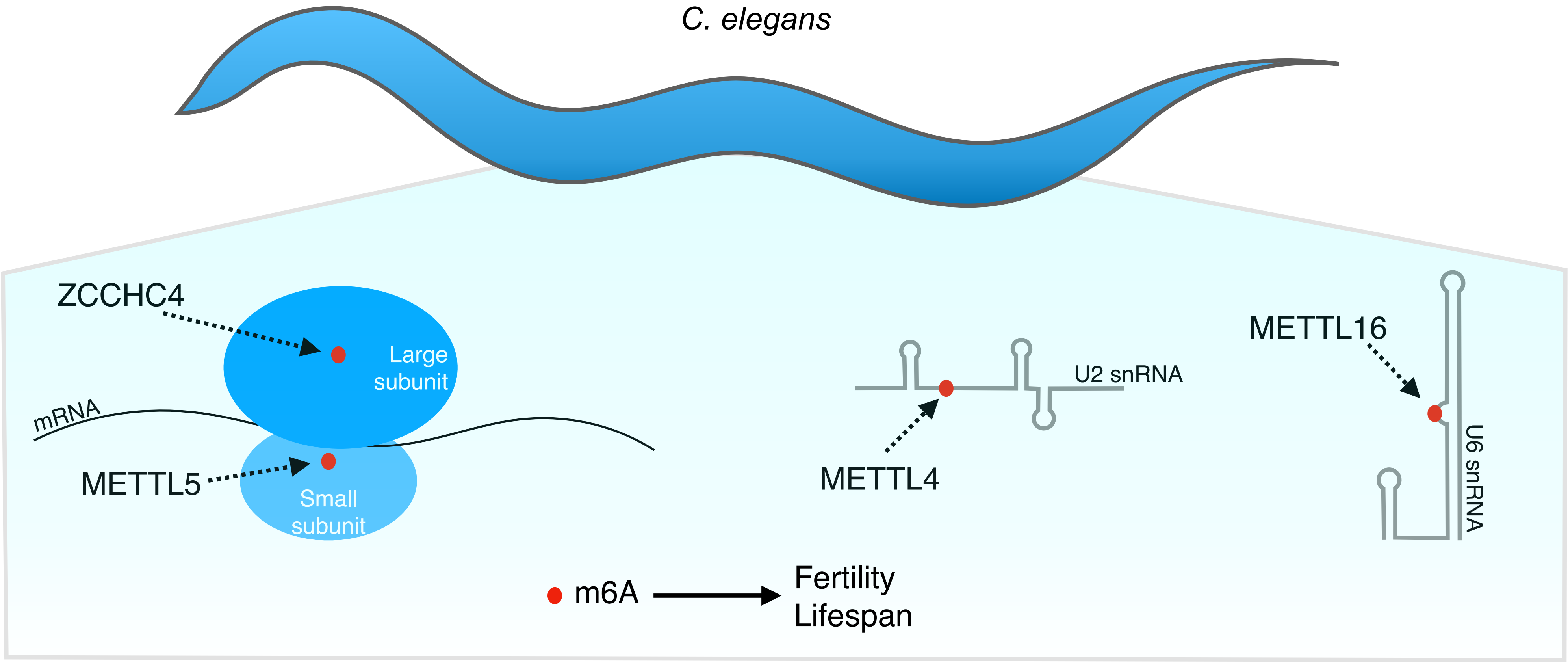

Supplement: Supplementary file 1 — Supplementary Information [file 41421_2020_186_MOESM1_ESM.pdf]
